# Route of exposure to veterinary products in bees: Unraveling pasture's impact on avermectin exposure and tolerance in stingless bees

**DOI:** 10.1093/pnasnexus/pgae068

**Published:** 2024-03-05

**Authors:** Diana Obregon, Olger Guerrero, David Sossa, Elena Stashenko, Fausto Prada, Beatriz Ramirez, Christophe Duplais, Katja Poveda

**Affiliations:** Department of Entomology, Cornell University, Ithaca, NY 14850, USA; New York State Integrated Pest Management Program, Cornell University, Geneva, NY 14456, USA; Department of Agronomic Engineering, La Salle University, Yopal, Casanare 850008, Colombia; Department of Entomology, Cornell University, Ithaca, NY 14850, USA; CROM-MASS Laboratory, Industrial University of Santander, Bucaramanga 680002, Colombia; CROM-MASS Laboratory, Industrial University of Santander, Bucaramanga 680002, Colombia; Department of Conservation and Environmental Sovereignty, ABC Colombia, Yopal, Casanare 850008, Colombia; Department of Entomology, Cornell AgriTech, Cornell University, Geneva, NY 14456, USA; Department of Entomology, Cornell University, Ithaca, NY 14850, USA

**Keywords:** pesticide exposure, pesticide tolerance, insecticides, veterinary products, biotransformation

## Abstract

Deforestation rapidly increases in tropical regions, primarily driven by converting natural habitats into pastures for extensive cattle ranching. This landscape transformation, coupled with pesticide use, are key drivers of bee population decline. Here, we investigate the impact of pasture-dominated landscapes on colony performance, pesticide exposure, and insecticide sensitivity of the stingless bee *Tetragonisca angustula*. We monitored 16 colonies located in landscapes with varying proportions of pasture. We collected bee bread for pesticide and palynological analysis. We found a positive correlation between pollen diversity and colony growth, with no effect of the proportion of pasture in the landscape. In contrast, we detected prevalent and hazardous concentrations of the insecticide abamectin (9.6–1,856 µg/kg) in bee bread, which significantly increased with a higher proportion of pasture. Despite the abamectin exposure, the bee colonies displayed no adverse effects on their growth, indicating a potential tolerance response. Further investigations revealed that bees from sites with higher proportions of pasture showed significantly reduced mortality when exposed to a lethal concentration of abamectin (0.021 µg/µL) after 48 h. Since abamectin is scarcely used in the study area, we designed an experiment to track ivermectin, a closely related antiparasitic drug used in cattle. Our findings uncovered a new exposure route of bees to pesticides, wherein ivermectin excreted by cattle is absorbed and biotransformed into abamectin within flowering plants in the pastures. These results highlight that unexplained exposure routes of bees to pesticides remain to be described while also revealing that bees adapt to changing landscapes.

**Significance Statement**

The decline of bees in agricultural environments is attributed to habitat loss and pesticide use, while the impact of livestock systems remains unclear. This study reveals that as cattle grazing pastures expand, bees are more exposed to the insecticide abamectin and develop an increased tolerance to it. The source of abamectin residues is traced back to the use of ivermectin, a related veterinary drug, applied to cattle. Ivermectin is excreted in animal feces, absorbed by flowering plants, and converted into abamectin through enzymatic reactions, ultimately collected by the bees. The findings indicate that landscape composition is pivotal in shaping pesticide tolerance and exposure among bees and address the risk of modified veterinary products in insect decline.

## Introduction

Grazing prairies cover 30–40% of total global land (1), and this area is rapidly increasing in the tropics and subtropics, mainly due to the conversion of primary and secondary forests into pastures for extensive cattle ranching (2–4). These land-use changes constitute the biggest drivers of pollinator loss and impairment of ecosystem function in the region (5). Furthermore, homogeneous landscapes are often associated with increased pesticide use (6), and although pesticides are commonly applied in livestock production systems (7), there is little information showing how veterinary uses of pesticides impact bees and pollinator communities (8).

Stingless bees are the most abundant and diverse group of bees in the tropics (9). These social bees play a vital role in pollinating many wild plants and crops (10, 11). Multiple studies show how the loss of natural habitat combined with agricultural intensification result in adverse effects on bees, such as exposure to lethal and sublethal doses of pesticides (12, 13), changes in diet composition (14), body size (15, 16), colony performance (17), and ultimately a decrease in bee populations (18, 19). However, the effect of expanding pastures for cattle on bees has not yet been well studied (20, 21). Here, we examine the impacts of livestock-dominated landscapes and the associated use of avermectins on colonies of the widespread stingless bee species *Tetragonisca angustula* (22).

Avermectins are a small family of natural products isolated from *Streptomyces avermitilis* since the 70s (23). They include the insecticide abamectin, which is also used as a veterinary anthelmintic, and the antiparasitic drug ivermectin, extensively used to control endo and ectoparasites in farmed animals. Abamectin differs from ivermectin by only one double bond in its chemical structure. Applications of avermectins are made with topical and injectable formulations and dip baths. The lack of technical training for farmers and the emergence of livestock pest resistance have exacerbated their use with little consideration for the environmental impacts (24). Abamectin and ivermectin have moderate to low toxicity in mammals and high toxicity in invertebrates, causing paralysis, starvation, and ultimately death (25). When ivermectin is administered to farm animals, it is metabolized by hydroxylation processes from the rumen, stomach, or intestine, with fecal excretion being the main route of elimination (26, 27). Subcutaneous applications of ivermectin in cattle showed residues in the feces of the animals up to 21 days postinjection, and the concentration of the product in the feces started at 87% of the administered concentration, progressively decreasing to 0.17% on day 21 (28). In addition, ivermectin diffuses into the soil from feces and is detected in nearby plants (29).

Ivermectin residues in feces have been shown to decrease the abundance and diversity of colonizing coprophagous insects (30–32) with potential additional negative effects on other groups of arthropods too (7, 33, 34). Recently, it was found that residues of avermectins, and other veterinary products used in cattle feed yards in the United States emanate into the environment polluting surrounding areas (35). Abamectin, but not ivermectin, has also been detected and quantified in wildflowers near feed yard boundaries where ivermectin has been used to treat cattle (36). Together, these studies suggest that cattle farming has the potential to expose bees to abamectin insecticides as a result of uptake and biotransformation (dehydrogenation) of ivermectin through flowering plants, which are pathways of widely underestimated exposure to pesticides for insects.

Research on pesticide toxicity and risk to bees has primarily emphasized on the development of acute toxicity essays in laboratory conditions for specific populations (37). However, less attention has been paid to how increased or continuous exposure to pesticides can change bee tolerance to pesticides and how this can be mediated by landscape composition. In pollen beetles (*Meligethes aeneus* Fabricius 1775), a major pest in oilseed rape, higher pesticide tolerant populations were found in landscapes with higher proportions of the crop (38), and given that these types of landscape effects are reported in different herbivores (39), it could be occurring in bees too. The counterintuitive idea that bees could adapt to certain types of pesticide exposure has yet to be explored. And, although bees have few detoxification genes compared to other insect genomes (40), pollen diet or landscape composition can potentially activate xenobiotic detoxification systems (41) leading to pesticide tolerance.

In this study, we investigate the impact of expanding pastures for cattle ranching on pesticide exposure and pollen diversity in the diet of *T. angustula* and how these factors affect colony growth and body size. Additionally, we explore whether *T. angustula* populations develop adaptive tolerance to the insecticide abamectin, stemming from increased exposure to ivermectin found in cow feces and converted into abamectin by flowering plants in the pastures. Overall, the research identifies a potential new route of abamectin exposure for stingless bee colonies.

## Results

In 2018, in Chameza, Casanare, a Colombian Andean town, we transferred 16 wild colonies of *T. angustula* to wooden hives and placed them next to the original locations. Colonies were naturally separated by a minimum distance of 1 km up to 28 km. The landscapes surrounding the colonies at a 500-m radius varied from 0.24 to 0.77 in their proportion of pasture area. During 4 months of the dry season (October 2018–January 2019), we collected beebread samples from the colonies to perform palynological and pesticide analysis. A total of 112 different pollen types were identified to the lowest taxonomic level possible (Table S1). The most frequent pollen types included Asteraceae*, Piper, Myrsine, Alchornea latifolia, Viburnum triphyllum, Rhynchospora nervosa, Hyptis, Toxicodendron striatum*, *Spermacoce,* and *Mimosa* (Fig. S2). We then calculated the Shannon diversity index for each bee bread sample at the morphotype level. Using ultrahigh liquid chromatography high-resolution mass spectrometry (UHPLC-HRMS), we screened for residues of 13 pesticides commonly used in agriculture and livestock production. These pesticides were identified based on personal interviews with local growers and cattle ranchers (Table 1). Eight of the pesticides tested were detected (methomyl, abamectin, bifenthrin, imidacloprid, cymoxanil, difeconazol, triclorfon, and propamocarb). Abamectin was the most prevalent pesticide, present in 59.3% of the samples, with concentrations ranging from 9.6 to 1856 µg/kg. Hazard quotient (HQ) calculations for all 13 molecules (13) showed that only abamectin raised lethal hazard concerns, particularly at the maximum concentration found. For body size estimation, we measured the intertegular distances of 10 worker bees per colony during the monthly sampling. Colonies were weighed when transferred to the hives and after every sampling date to calculate the monthly change in weight, subsequently called colony growth.

**Table 1.** Frequency, range, mean, limit of quantification, and HQ of pesticide residues detected in beebread samples of *T. angustula* colonies in Chameza, Casanare, Colombia.

Use/Type	Pesticide tested	Frequency of samples with pesticides detected (%) *n* = 48	Concentration range of quantified samples (ng/g)	Mean conc. (ng/g)	Limit of quantification (ng/g)	HQ (based on mean conc.)	HQ (based on max. conc.)
*Agriculture*							
Insecticide	Methomyl	18.7	0.1–1	0.26	0.1	0	0
Insecticide	Abamectin	59.3	9.6–1856	351.3	8	28	**147.7**
Insecticide	Bifenthrin	3.1	14.1	14.1	0.4	0.1	0.1
Insecticide	Imidacloprid	3.1	0.7	0.7	0.1	0.1	0.1
Insecticide	Profenofos	_	_	_	0.5	_	_
Insecticide	Lufenuron	_	_	_	0.8	_	_
Fungicide	Cymoxanil	6.2	0.1–0.3	0.2	0.1	_	_
Fungicide	Difenoconazole	6.2	0.2–39.4	19.8	0.2	_	_
Fungicide	Propamocarb	21.8	0.1–1.8	0.6	0.1	_	_
*Livestock*							
Insecticide	Doramectin	_	_	_	21	_	_
Insecticide	Ivermectin	_	_	_	8	_	_
Insecticide	Cypermethrin	_	_	_	8	_	_
Insecticide	Trichlorfon^a^	3.1	16	16	8	_	_

HQ ≥ 100 indicates these levels of pesticide exposure have reached ≥100% of honey bee LD50 levels, constituting a lethal hazard for bees. _, Not detected. ^a^Oral LD for trichlorfon was not found.

### Landscape effects on colony growth and body size mediated by pollen diversity and pesticide exposure

**Fig. 1.**
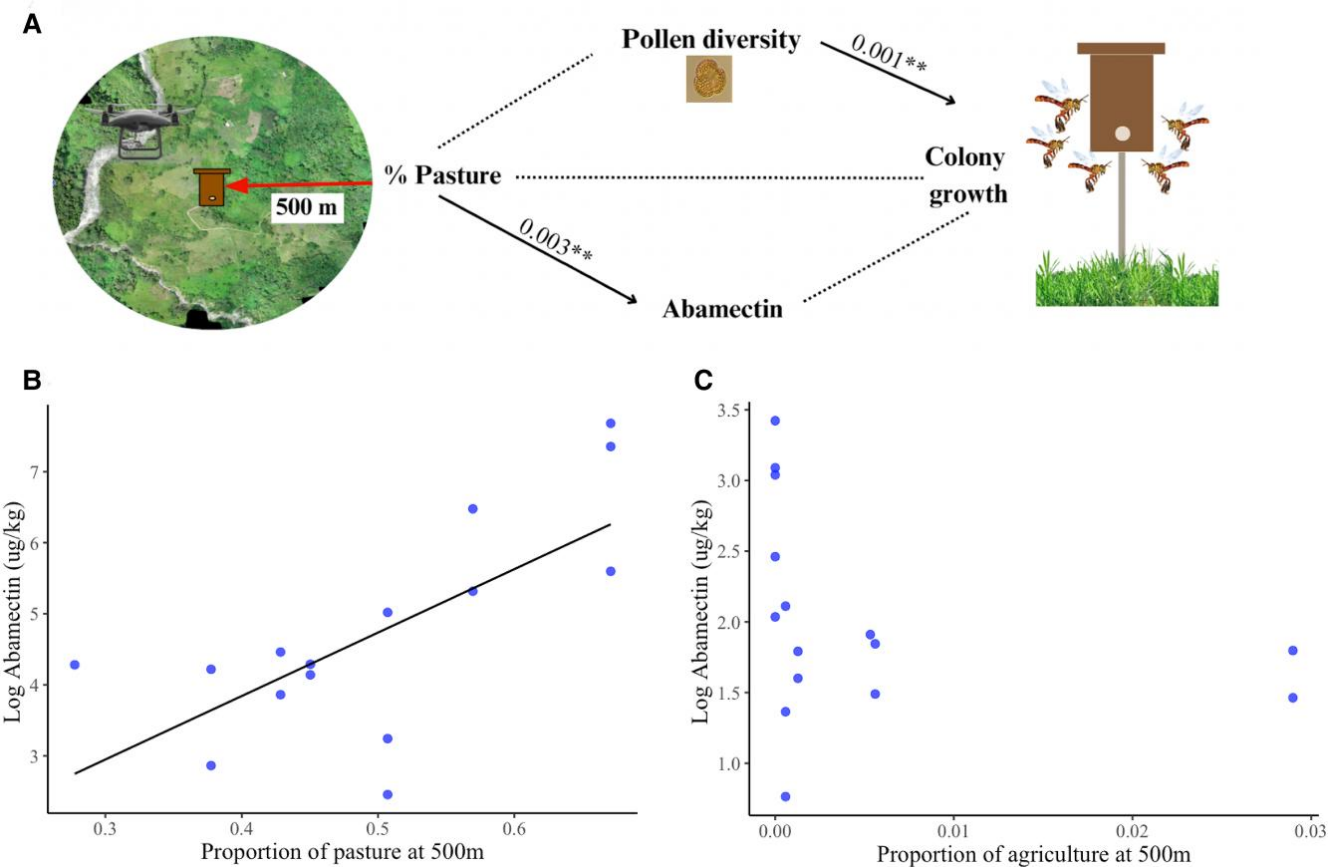
A) Path model analysis of the relationships between the proportion of pasture in the landscape at 500 m, pollen diversity in the bee bread (Shannon index), and colony growth of 16 colonies of *Tetragonisca angustula* bees located in Chameza, Casanare, Colombia. The number along the arrows are the *P*-values and stars demark the significance level (*P < 0.05, **P < 0.01, ***P < 0.001). B) Average log concentration of abamectin residues found in three monthly samplings of bee bread in 16 *T. angustula* colonies in different sites varying in the proportion of pasture area at 500 m around the colonies. The solid line indicates a significant effect. C) Average log concentration of abamectin residues found in three monthly samplings of beebread samples in 16 *T. angustula* colonies in different sites varying in the proportion of agricultural area at 500 m around the colonies.

We conducted a path analysis to evaluate the direct and indirect effects of the proportion of pasture on colony growth through changes in pollen diversity (Shannon index) and abamectin residues. The results of Shipley's test of d-separation supported the causal assumptions in the path model, indicating that they provided a good fit to the data (Fisher's *C* = 0.468, df = 2, *P* = 0.791, Fig. 1A). Surprisingly, we did not find a direct or indirect effect of pasture on colony growth. However, we noticed a notable correlation: an increase in the proportion of pasture at 500 m led to a rise in abamectin concentration in beebread (GLM_quassipoisson_ Pasture estimate = 8.4, *t*(_1,34_) = 3.34, *P* = 0.0021, Fig. 1B), indicating heightened pesticide exposure in sites with more grazing areas. We also conducted a similar path analysis substituting colony growth with the intertegular distance as the response variable. None of the variables on this path analysis significantly affected intertegular distance (Fig. S3). To rule out that the abamectin residues came from neighboring crops, we evaluated the relationship between abamectin and the proportion of agriculture in the landscape and did not find a significant effect (GLM_quassipoisson_ Agriculture estimate *= −*85.7, *t*_(1,34)_ = −1.69, *P* = 0.09, Fig. 1C).

### Tolerance of *T. angustula* to abamectin

**Fig. 2.**
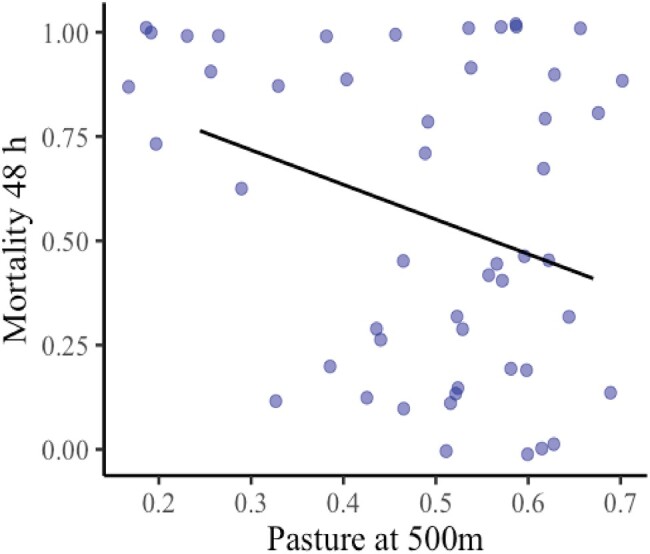
Proportion of mortality at 48 h of *Tetragonisca angustula* adult worker bees fed with a solution of sugar water containing a discriminatory concentration of abamectin based on the oral LC50 (0.021 µg/µL) in relation to the proportion of pasture area at 500 m around the colonies of origin of the bees (11 colonies). Every dot represents the mortality of a cup with 10 worker bees. The black line denotes a statistically significant relationship (*P* < 0.05).

In light of our discovery of abamectin residues in colonies, which increased with higher pasture proportions in the landscape without affecting colony growth or body size, we sought to explore whether variations in exposure between sites might lead to changes in bee tolerance to abamectin as a possible adaptive response to pesticide exposure. To test this hypothesis, we calculated a lethal oral concentration used as a discriminatory dose (oral LC50 = 0.021 µg/µL) (Table S4, Fig. S5) to feed 50 adult worker bees from 12 sampling sites. Bees from every site were randomly distributed in five (150 mL) deli cups with ten bees per cup. Mortality was recorded at 24 and 48 h. At 24 h, the mortality was not affected by the proportion of pasture in the landscape (binomial glmm: Pasture estimate: −1.27, *z* = −0.375, *P* = 0.708, Fig. S6). However, at 48 h, we found decreased mortality in bees from sites with higher pasture proportions, indicating an increased tolerance in those landscapes (binomial glmm: Pasture estimate: −4.048, *z* = −1.973, *P* = 0.0485, Fig. 2).

### Avermectin exposure route

**Fig. 3.**
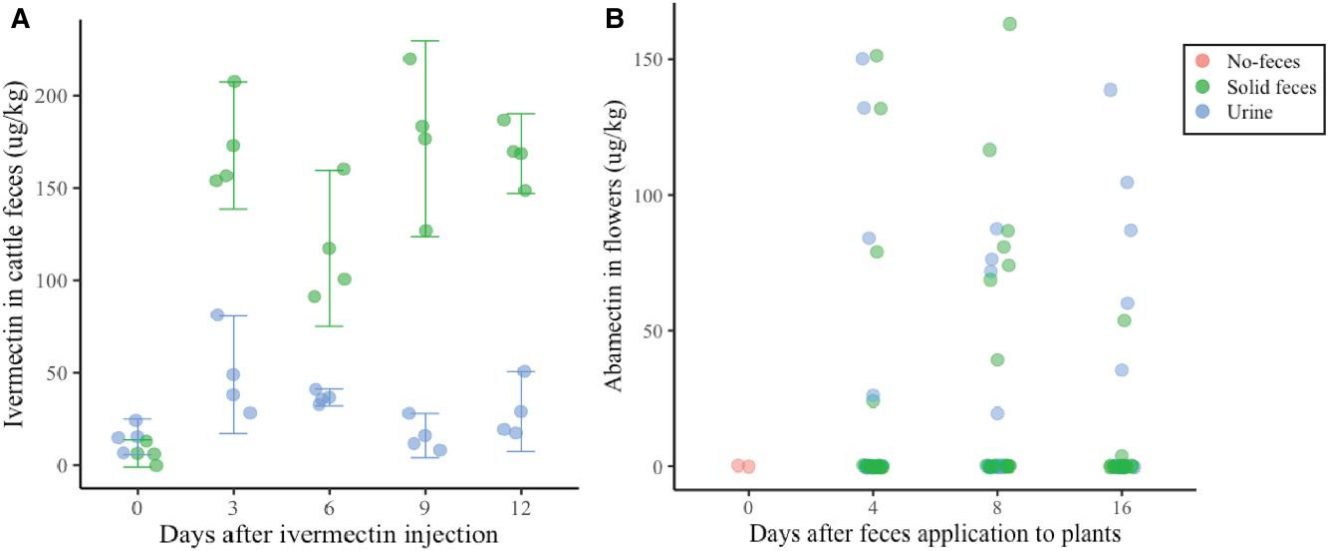
A) Ivermectin residues found in feces and urine samples from Cebu Brahman cattle treated with the commercial product Ivermectina 3.15%. No abamectin residues were detected in the excretions. B) Abamectin residues found in flowers of *Vernonanthura patens* after receiving applications of feces and urine samples contaminated with ivermectin. No ivermectin residues were detected in flowers.

As we reveal a correlation between the presence of abamectin residues in colonies and the proportion of pasture in the landscape, rather than the proportion of agriculture, and that abamectin is not frequently used in cattle ranching in this region, we wanted to further investigate the potential origin of these residues. We hypothesized that the abamectin found in bee bread could be the result of ivermectin biotransformation into abamectin within the flowering plants. This conversion through desaturation is a common enzymatic reaction observed in various plant families including Asteraceae (42, 43). We predicted that cattle treated with ivermectin, excrete ivermectin that are taken up by flowering plants in pastures leading to the presence of abamectin in the flowers after conversion by desaturase. To test this hypothesis, we designed an experiment where a commercial antiparasitic product with ivermectin (3.15% i.a.) was injected to 16 adult cows at the recommended dose. Urine and feces samples were collected at different time points, before the injection (day 0), and 3, 6, 9, and 12 days after the application. These excretions were applied to wildflower patches in a nongrazed prairie around the stems of *Vernonanthura patens*, a common Asteraceae in the area visited by *T. angustula*. Analysis using UHPLC-MS detected ivermectin residues in the cattle excretions with higher concentrations in the solid feces (128 µg/kg ± 18.6, mean ± SE) compared to the urine (29.2 µg/kg ± 4.9, mean ±SE) (Fig. 3A). No abamectin residues were detected in the excretions. As for flowers, we found abamectin residues after both urine (29 µg/kg ± 6, mean ± SE) and feces applications (24.38 µg/kg ± 5.5, mean ± SE) and no ivermectin residues were detected (Fig. 3B).

**Fig. 4.**
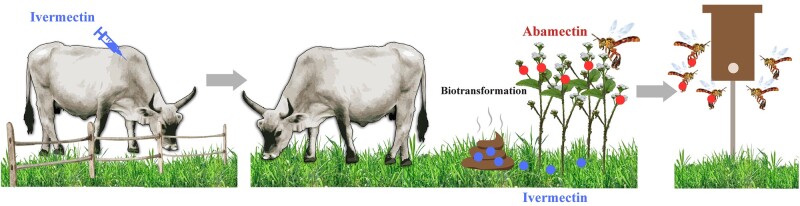
Potential route of avermectin exposure in stingless bee colonies located in livestock-dominated landscapes. When ivermectin is applied to cattle to treat endo and ectoparasites, residues end up in the urine and fecal feces, which flowering plants can absorb. Inside the plant, ivermectin can biotransform into abamectin through desaturase enzymes (*FAD*2 gene) and contaminate the pollen. Bees forage contaminated pollen that is transported to their colonies.

Based on these findings, we propose the following pesticide exposure route for bees. When ivermectin is applied to cattle, residues remain in the excretions. During cattle grazing in pastures, these contaminated feces and urine can be absorbed by flowering plants. Once inside the plant, an oxidation process, specifically a desaturation enzymatic reaction transforms ivermectin into abamectin residues, also understood as ivermectin metabolites (Fig. S7). These abamectin residues end up present in the pollen, collected by bees, and eventually stored in the bee bread as evidenced in this study (Fig. 4).

## Discussion

In this study, the impact of increasing pasture area for cattle grazing on colony growth and bee body size in *T. angustula* populations was explored. Surprisingly the expected effects of pasture on bee traits mediated by pollen diversity and pesticide exposure were not supported by the data. However, four unexpected findings emerged. Firstly, hazardous concentrations of abamectin were prevalent in *T. angustula* beebread. Secondly, there is a positive correlation between the proportion of pasture areas in the landscape and the abamectin concentration. Thirdly, bees from these livestock-dominated landscapes exhibited an augmented tolerance to abamectin in a mortality bioassay. Lastly, the potential route of exposure for abamectin to reach colonies seemed to originate from ivermectin applications in cattle farms. We suggest that desaturase enzymes in Asteraceae biotransform ivermectin into abamectin within the plants leading to abamectin exposure when bees are foraging the flowers. The abundance of desaturase enzymes (*FAD*2 gene) in plants (43) highlights the significance of recognizing that the biotransformation observed in this study, as also suggested by a previous study (36), is widespread in pastures worldwide and is not restricted to the specific study area. These striking results reinforce the argument that pesticide risk assessments must extend cropland areas and consider the potential contribution of animal production systems to pesticide exposure and changes in pesticide sensitivity within bee populations.

The absence of direct or indirect effects of the proportion of pasture on colony weight and intertegular distance can be a result of the plasticity and generalist feeding behavior of *T. angustula* (44). Pastures in this region consist of a mix of Poaceae and Cyperaceae plants alongside other wildflower patches, providing enough floral resources to fulfill the nutrition requirements of this bee species. Moreover, we did not find a relationship between the proportion of pasture area and pollen diversity. Previous studies in bumble bees (45) and honey bees (46) have also shown a lack of landscape composition effect on pollen diversity in the colonies, suggesting that social bees can compensate for limited floral resources in the vicinities by expanding their foraging range to ensure sufficient and diverse pollen intake (47). Additionally, an independent effect was found. An increase in pollen diversity was associated with an increase in colony weight, reiterating that stingless bees need access to a diverse diet to maintain their offspring throughout the year. This result aligns with work on bumble bees located in maize-dominated landscapes, where reduced pollen diversity led to a decline in colony growth (48).

Previous studies have detected avermectin residues in wildflowers and wild bees surrounding cattle feed yards (36, 49), and the present work now reveals that abamectin in bee colonies can be directly linked to the area of grazed pastures or to sites where cattle excrements are present. This effect of land use on pesticide exposure has previously been tested in agricultural settings with mixed evidence. For instance, in apple orchards, an increasing proportion of agriculture in the landscape increased the levels of fungicide risk in the pollen provisions of *Osmia cornifrons* (50). However, in the case of honey bees immersed in a heterogeneous agricultural matrix, landscape composition had no effect on pesticide levels (51). These discrepancies might arise due to variations in foraging strategies, flight range capacities between species, and various patterns of local pesticide use. Although we did not find a negative effect of abamectin on colony growth or intertegular distance, further research is needed to explore the impact on other aspects such as foraging capacity, learning, and reproduction.

The reduced mortality and consequently increased tolerance among worker bees from sites with high proportion of pastures and greater exposure to avermectins was compelling. To the best of our knowledge, this discovery marks the first instance of pesticide tolerance levels in bees regulated by the landscape, prompting further investigation into how landscape composition and continuous pesticide exposure influence bee pesticide tolerance. Previous evidence suggests that bees exposed to sublethal doses of pesticides might develop an increased tolerance to these compounds due to upregulation of detoxification and autoimmune genes (52, 53). Another study on honey bees exposed to imidacloprid demonstrated a context-dependent resilient response, with free-foraging bees showing higher detoxification gene expression cytochrome P450 compared to caged bees (54). Continuous exposure to sublethal doses of avermectins, combined with certain pollen diets could contribute to the reduced mortality of *T. angustula* populations, but more research is needed to understand the underlying mechanisms and consistency of this pattern. Additionally, it is crucial to evaluate if this response is inheritable and qualifies as resistance, as well as to assess potential adaptation costs. Reductions in longevity and reproduction are commonly found in insect pesticide resistance studies (55).

Our study uncovers a novel pesticide exposure route for *T. angustula* colonies. Ivermectin residues present in cattle excretions can contaminate plants in pastures, leading to abamectin residues in the flowers that bees use to forage pollen. Previously, it has been demonstrated that ivermectin in feces can move to the soil and be absorbed by nearby plants (29). And although ivermectin is produced from abamectin by reducing a double bond at positions 22 and 23 (56), other oxidation processes such as oxidation of alcohol into ketone by microorganisms in the soil (57) or enzymes in plants (58) can form abamectin-like metabolites yet to be discovered (59).

This work, which indicates increased abamectin exposure and higher tolerance in bees from livestock-dominated landscapes, underscore the necessity for further ecotoxicological and risk assessment studies regarding bees’ responses and potential adaptations to veterinary drugs used in animal husbandry. This aligns with previous findings of pesticides detected in rivers in the United Kingdom associated with flea treatments used for domestic animals (60). Regulatory agencies should expand their considerations beyond the impact of agrochemicals on pollinators and include the environmental fate and effects of animal medications through soil, water, and other routes of exposure on various organisms.

## Methods

### Study sites

Our main location was the municipality of Chameza (Casanare) in the eastern Andean Cordillera in Colombia. Here, we performed all the components of the path analysis, including landscape characterization, pollen and pesticide sampling, colony growth, and intertegular distance metrics as well as the mortality bioassay. This region is dominated by mountains with steep slopes, with all our sites located in a height range of 1,188 to 1,683 masl. The dominant land covers are primary and secondary forests, pastures (grazing lands), and small-scale crop areas with *Solanum quitoense*, sugar cane, and plantain. To determine the abamectin oral LC50 for *T. angustula,* we collected adult worker bees from the bee preserve AYNI in La Mesa, Cundinamarca (4°41′39″N, 74°25′48″W), also located in the eastern Andean Cordillera in Colombia. We selected a different study site with similar geography to ensure the bees came from a place with no pesticide exposure and >75% percent natural habitat in the landscape. For the pesticide exposure route experiment, we selected two sites in Yopal, Casanare, a livestock farm (5°12′4.68″N, 73°3′0.3″W) with easy terrestrial access, and a private grassland reserve “El tiestal” (5°18′17.4″N, 72°10′53.18″W), where farm animals have not grazed in the past 10 years. Yopal, is a municipality near Chameza, dominated by grazing lands in flooded savannahs and similar livestock practices.

### Landscape characterization and bee colony transfers

In July 2018, with the help of local farmers, we located 16 wild colonies of *T. angustula* in different villages that were transferred to equally sized wooden hives at the exact same place. Using a DJI Phantom 4 drone, and the *Map Pilot* application, we acquired high-resolution images (5 cm/pixel) at 500 m around each colony based on the estimated flight range for *T. angustula* (61). Images per site were combined in orthophotos in which polygons of the different land cover (natural habitat, pastures, and agriculture) were drawn manually to calculate the area and the proportions of the land covers using QGIS (62). The sites were separated by a minimum distance of 1 km up to 28 km. Among types of land covers, the proportion of natural habitat and the proportion of pasture were highly correlated (Pearson's *r* = −0.97, *P* < 0.001, *n* = 16), and together comprised most of the landscape area around each farm (87–99%). We used pasture as the landscape explanatory variable based on this close correlation. The pasture proportion among sites ranged from 0.24 to 0.77.

### Colony growth and body size

After the colonies were well adapted to the hives, we sampled them in October, November, December 2018, and January 2019 during the dry season. At each sampling date, we weighted the whole hive and collected 10 worker bees leaving the colony in ethanol (85%) to measure intertegular distance as an index of body size. The proportional differences of colony weights among samplings were used as the response variable for colony performance, and it is called in this manuscript colony growth.

### Palynological analysis

During each monthly sampling, we extracted 5 g of bee bread from 10 recent pollen pots inside the colonies to identify the pollen types. The samples were acetolized following the method of Erdtman (63). A permanent slide was mounted per each sample and then observed under an optical microscope (40×) for pollen type identification and pollen counting. Transects were initiated at a random location on the margin of each slide, and all pollen grains that were entirely in the field of view were counted and classified until a minimum of 300 pollen grains total was reached. Pollen was identified to the lowest taxonomic level possible by direct comparisons with the reference pollen collection of the Bee Research Laboratory LABUN at the National University of Colombia, as well as using the pollen catalogs of Roubik and Moreno (64), Montoya-Pfeiffer et al. (65), Giraldo et al. (66), and PalDat (67). Based on these results we calculated the Shannon diversity index (68) to estimate the pollen diversity per sample.

### Pesticide analysis

We collected 16 beebread samples (1 g each) at three-time points during November, December, and January from 10 recent pollen pots inside the colonies to perform pesticide analysis in the Laboratory of Chromatography and Mass Spectrometry CROM-MASS at the Universidad Industrial de Santander. Samples were tested for nine pesticides commonly used in agriculture in the region: methomyl, abamectin, bifenthrin, imidacloprid, profenofos, lufenuron, cymoxanil, difenoconazole, propamocarb, and five pesticides commonly used in livestock production: ivermectin, doramectin, triclorphon, and cypermethrin. Samples were mashed in liquid nitrogen followed by the extraction with the QuEChERS method. The extractions were analyzed UHPLC with an *Dionex Ultimate 3000* (Thermo Scientific) equipped with a binary bomb of gradient (HP G3400RS), an automatic injector of samples (WPS 300TRS) and a thermoset unit for the column (TCC 3000). The LC-MS interface was electronebulization and a high-resolution mass spectrometer with a detection system of ions *Orbitrap*. Chromatographic separation was made with a column *Hypersil GOLD Aq* (Thermo scientific, 100 × 2.1 mm, 1.9 μm particle size) at 30 °C. The mobile face was A: Aqueous solution 0.2% formic acid and ammonium formate 5 mM and B: acetonitrile with 0.2% formic acid and ammonium formate 5 mM. The initial condition of the gradient was 100% A, changing linearly until 100%B (8 min), it remained 4 min, with a return to the initial condition in 1 min. The total run time was 13 min, with 3 min after the run. The mass spectrometer Orbitrap (Exactive Plus, Thermo Scientific) was connected to the electronebulization interface (HESI), and operated in positive mode with a capillar voltage of 4.5 kV. Nitrogen was used as drying gas. The mass spectrum was acquired in the mass range 60–900 *m*/*z*. The Orbitrap mass detector was calibrated with the certified reference solutions: Ultramark 1621 Mass Spec. (AB172435, ABCR GmbH & Co. KG), sodium dodecyl sulfate (L45509, Sigma-Aldrich), and sodium taurocholate hydrated (T4009, Sigma-Aldrich). Compound identification was made using the acquisition mode *Full scan* and the extraction of ions corresponding to the pesticides tested [M + H]^+^ or [M + Na]^+^ with mass accuracy of Δ_ppm_ <3 and using a mix standard solution of the pesticides. Abamectin is a mix between Avermectin B_1a_ (C_48_H_72_O_14_ Exact Mass: 872.4922) and Avermectin B_1b_ (C_47_H_70_O_14_, Exact Mass: 858.4766). Ivermectin is a mix between Ivermectin B_1a_ (C_48_H_74_O_14_ Exact Mass: 874.5073) and Ivermectin B_1b_ (C_47_H_72_O_14_, Exact Mass: 860.4917). Concentrations of avermectin and ivermectin were calculated by summing Avermectin B_1_ and Avermectin B_1b_ concentrations and Ivermectin B_1a_ with Ivermectin B_1b_ concentrations, respectively.

Based on the pesticide residues found in the bee bread, we estimated the pesticide HQ following this formula (13):


$$\begin{array}{r} \mathrm{Hazard}\;\mathrm{quotient}(\text{\%}\mathrm{Oral}\;\mathrm{LD}50)=\frac{\mathrm{Concentration}\;\mathrm{of}\;\mathrm{the}\;\mathrm{pesticide}\;\mathrm{found}\;[\mathrm{ng}/g]\times \mathrm{Amount}\;\mathrm{of}\;\mathrm{bee}\;\mathrm{bread}\;\mathrm{consumption}\;\mathrm{by}\;\mathrm{larvae}}{\mathrm{Oral}\;\mathrm{LD}50\;[\mathrm{ng}/\mathrm{bee}]}\times 100 \end{array}.$$

The concentrations of the residues in bee bread are reported as nanograms of active ingredient per gram (ng a.i./g), the amount of exposure to the matrix (beebread) in g/bee, and the LD50s in ng a.i./bee (LD50: Lethal doses causing 50% mortality). The amount of exposure to bee bread for *T. angustula* was obtained from previous calculations made by Dorigo et al. (69). The estimated amount of pollen consumed by a larva is 0.00796 g of larval food/cell. For adult consumption, there is no estimate available for the amounts of pollen it can consume. We used honey bee oral LD50s values given that there is no information for *T. angustula* for all the pesticides. Oral LD50s were obtained from the ECOTOX database of the US-Environment Protection Agency (http://cfpub.epa.gov/ecotox/) when available, data for methomyl from Clinch (70), abamectin from Del Sarto (71), and imidacloprid from Nauen (72). An HQ ≥ 100 indicates these levels of pesticide exposure can cause ≥50% mortality in honey bees, constituting a lethal hazard.

### Path analysis

To evaluate the direct and indirect effects of pasture on colony growth and intertegular distance through pollen diversity and pesticide residues, we conducted a path analysis using the piecewiseSEM package (73). Since abamectin was the most frequent and concentrated insecticide detected and is related to ivermectin, a highly used veterinary insecticide in the area, we used it as a variable in the path analysis. In the path models, we used generalized linear models fitting Shannon index, colony growth, and intertegular distance with Gamma error distribution and link inverse and abamectin residues with quasipoisson error distribution and link log. We used quasipoisson to account for overdispersion. The overall fit of the path model was tested using Shipley's d-separation test for each possible independent claim, and Fisher's C statistics to test whether the observed levels of correlation across all independent claims can be explained by random variation.

### Sensitivity of bees to abamectin in a landscape gradient of exposure

We calculated the abamectin LC50 at 24 h (0.021 µg/µL) for *T. angustula* bees from six nonpreviously exposed colonies to pesticides as a discriminatory dose to evaluate whether there is a differential sensitivity along the landscape/exposure gradient (see supplements for LC50 calculation, Table S8). From 11 of the characterized sites, we collected about 50 adult worker bees emerging from the colonies that were randomly distributed in five 150 mL deli cups, one cup with 10 bees was the control treatment where the bees were fed ad libitum with 1:1 sucrose:water only, and the rest were distributed in four cups and fed with the discriminatory lethal dose concentration in a solution of 1:1 sucrose:water. Mortality was recorded at 24 and 48 h. The results were analyzed using GLMM with site as a random effect and a binomial distribution with link logit.

### Abamectin/ivermectin exposure route experiment

#### Cattle treatment with ivermectin

The livestock production system in the study area is extensive with cattle moving across the prairies for grazing. However, pesticide applications are centralized, ranchers gather the animals in a pen that is usually at the center of the farm to inject or bathe the animals with the products. To determine whether an increased abamectin exposure can be related to the use and metabolization of ivermectin as a veterinary pesticide, we wanted to test if the feces and urine from cattle treated with ivermectin can contain residues that can be later absorbed by plants in the grazing areas, reaching the nectar and pollen of wildflowers that bees collect.

In February 2020, on a cattle ranching farm in Yopal, Casanare, Colombia (5°12′4.68″N, 73°3′0.3″W), we selected 16 Cebu Brahman cows (5 to 8 years old), farmed for meat production, that had not been treated with any insecticide in the last year. The animals were gathered in a pen previously covered with a layer of black polyethylene (caliber 60), installed to avoid contact with the ground and to be cleaned between samples. The animals were left in the pen for a couple of hours and at the end, we collected feces and urine samples from the plastic layer before the application of the insecticide. Subsequently, the animals were weighed one by one and injected intramuscularly with 1 mL per every 50 kg of body mass with the commercial insecticide Ivermectina 3.15%. Three, 6, 9, and 12 days after injections, the animals were re-gathered in the pen to collect urine and feces samples. Four 500 g of feces and four of 250 mL samples of urine were collected at each time point. Samples were frozen at −20 °C while the second part of the experiment began.

#### Wildflowers exposed to cattle excretions

In Yopal, Casanare, in a private grassland reserve “El tiestal” (5°18′17.4″N, 72°10′53.18″W), that has not been grazed by farm animals in the past 10 years, we selected wildflowers of the species *Vernonanthura patens* that are commonly found in commercial ranching farms and are frequently visited by stingless bees and solitary bees. We applied to the soil the feces and urine samples collected from the different collection days on the root area's surface. With four patches per type of excretion (feces and urine) and day of collection (0, 3, 6, 9, 12) for a total of 40 plants treated. Ten recently opened flowers of the plants were then collected 4, 8, and 16 days after application. One gram of the feces, 3 mL of urine, and five flowers per plant were analyzed using UHPLC (the same methods described previously) to look for abamectin and ivermectin residues.

## Supplementary Material

pgae068_Supplementary_Data

## Data Availability

All data are included in the manuscript and supplementary material.
