# Supplementary material for: Route of exposure to veterinary products in bees: Unraveling pasture's impact on avermectin exposure and tolerance in stingless bees
Source: PNAS Nexus. 2024 Mar 5;3(3):pgae068. doi: 10.1093/pnasnexus/pgae068 (PMC10914370; doi:10.1093/pnasnexus/pgae068)
Supplement: pgae068_Supplementary_Data [file pgae068_supplementary_data.zip › PNASNEXUS-PNASNEXUS-2023-00846R-s06.docx]

**Supplementary information:**

**Route of exposure to veterinary products in bees:**

**unraveling pasture’s impact on avermectin exposure and tolerance in stingless bees.**

Diana Obregon^1,2^, Olger Guerrero^3^, David Sossa^1^, Elena Stashenko^4^, Fausto Prada^4^, Beatriz Ramirez^5^, Christophe Duplais^6^, Katja Poveda^1^

1. Department of Entomology, Cornell University, Ithaca, NY, USA, 14850.
2. New York State Integrated Pest Management Program, Cornell University, Geneva, NY, 14456, USA.
3. Department of Agronomic Engineering, La Salle University, Yopal, Casanare, 850008, Colombia.
4. CROM-MASS Laboratory, Industrial University of Santander, Bucaramanga, 680002, Colombia.
5. ABC Colombia, Yopal, Casanare, 850008, Colombia.
6. Department of Entomology, Cornell AgriTech, Cornell University, Geneva, NY, 14456, USA.

*Corresponding author: Diana Obregon

[do265@cornell.edu](mailto:do265@cornell.edu)

**Table S1.** Frequency and average proportion of pollen types found in *Tetragonisca angustula* beebread in Chameza, Casanare, Colombia

| **Frequency in the samples (%)** | **Average % in the samples** | **Family** | **Genus or species** |
| --- | --- | --- | --- |
| 97.7 | 14.256989 | Asteraceae | *Baccharis* |
| 94.2 | 18.856997 | Piperaceae | *Piper* |
| 88.4 | 4.053575 | Myrsinaceae | *Myrsine sp.* |
| 81.4 | 4.899483 | Euphorbiaceae | *Alchornea latifolia* |
| 77.9 | 16.590901 | Adoxaceae | *Viburnum triphyllum* |
| 74.4 | 5.078553 | Cyperaceae | *Rhynchospora nervosa* |
| 73.3 | 2.235363 | Lamiaceae | *Hyptis type* |
| 57.0 | 5.662476 | Anacardiaceae | *Toxicodendron striatum* |
| 53.5 | 0.579244 | Rubiaceae | *Spermacoce* |
| 45.3 | 2.338535 | Fabaceae | *Mimosa pudica* |
| 44.2 | 2.515771 | Muntingiaceae | *Muntingia calabura* |
| 44.2 | 0.969929 | Asteraceae | *Steiractinia type* |
| 43.0 | 2.870117 | Escalloniaceae | *Escallonia pendula* |
| 43.0 | 0.36364 | Fabaceae | *Cassia type* |
| 40.7 | 0.712766 | Cannabaceae | *Trema* |
| 40.7 | 0.279124 | Asteraceae | *Emilia* |
| 38.4 | 0.499492 | Begoniaceae |  |
| 36.0 | 1.900488 | Apiaceae | *Spananthe paniculata* |
| 34.9 | 0.255841 | Amaranthaceae | *Alternanthera* |
| 33.7 | 0.553974 | Malvaceae | *Heliocarpus* |
| 32.6 | 0.65702 | Asteraceae | *Vernonanthura* |
| 32.6 | 0.249552 | Asteraceae | *Elephantophus/Eirmocephala brachiata* |
| 31.4 | 3.128772 | Melastomataceae | *Miconia* |
| 30.2 | 0.192526 | Poaceae |  |
| 26.7 | 0.392874 | Fabaceae | *Erythrina* |
| 26.7 | 0.180701 | Rhamnaceae | *Gouania polygama* |
| 24.4 | 0.52034 | Myrtaceae | *Psidium* |
| 24.4 | 0.208692 | Loranthaceae | *Orycthanthus* |
| 23.3 | 0.3243 | Melastomataceae |  |
| 22.1 | 0.640068 | Solanaceae | *Solanum quitoense* |
| 22.1 | 0.166747 | Fabaceae | *Gliciridia sepium* |
| 20.9 | 0.103324 | Polygonaceae | *Polygonum* |
| 19.8 | 0.148697 | Urticaceae | *Cecropia* |
| 17.4 | 0.191815 | Fabaceae | *Mimosa caesalpiniifolia* |
| 15.1 | 0.318122 | Malvaceae | *Theobroma cacao* |
| 15.1 | 0.115748 | Chenopodiaceae |  |
| 14.0 | 0.085532 | Sapindaceae |  |
| 14.0 | 0.073242 | Lithraceae | *Cuphea racemosa* |
| 12.8 | 0.657847 | Euphorbiaceae | *Acalypha* |
| 12.8 | 0.105783 | Araliaceae | *Oreopanax* |
| 12.8 | 0.050503 | Boraginaceae | *Cordia* |
| 11.6 | 0.064966 | Polygonaceae | *Triplaris americana* |
| 11.6 | 0.037634 | Acanthaceae |  |
| 10.5 | 0.093463 | Solanaceae | *Solanum quitoense* |
| 10.5 | 0.035372 | Myrtaceae | *Syzygium* |
| 9.3 | 0.97252 | Euphorbiaceae | *Sapium* |
| 9.3 | 0.076167 | Sapindaceae |  |
| 9.3 | 0.064089 | Primulaceae | *Ardisia* |
| 9.3 | 0.061202 | Chenopodiaceae |  |
| 9.3 | 0.048544 | Melastomataceae | *Miconia* |
| 8.1 | 0.862758 | Fabaceae | *Gliciridia sepium* |
| 8.1 | 0.247214 | Hypericaceae | *Vismia* |
| 8.1 | 0.217908 | Moraceae |  |
| 7.0 | 0.218011 | Fabaceae |  |
| 7.0 | 0.192787 | unknown |  |
| 7.0 | 0.160666 | Solanaceae |  |
| 7.0 | 0.159279 | Malphigiaceae |  |
| 7.0 | 0.158755 | Solanaceae |  |
| 7.0 | 0.085261 | Fabaceae |  |
| 7.0 | 0.084494 | Asteraceae |  |
| 7.0 | 0.072541 | Lithraceae | *Adenaria floribunda* |
| 7.0 | 0.057277 | Burseraceae | *Protium* |
| 5.8 | 0.18975 | Fabaceae |  |
| 5.8 | 0.058686 | unknown |  |
| 5.8 | 0.024534 | Fabaceae |  |
| 5.8 | 0.021561 | Amaranthaceae | *Alternathera* |
| 5.8 | 0.019449 | Sapindaceae |  |
| 5.8 | 0.015361 | Malvaceae | *Corchorus orinocensis* |
| 5.8 | 0.012877 | unknown |  |
| 4.7 | 0.36275 | Fabaceae |  |
| 4.7 | 0.272562 | Cyperaceae |  |
| 4.7 | 0.04506 | Polygonaceae |  |
| 4.7 | 0.036196 | Boraginaceae | *Cordia* |
| 4.7 | 0.022197 | Solanaceae |  |
| 4.7 | 0.021371 | Fabaceae |  |
| 4.7 | 0.020955 | Convolvulaceae |  |
| 4.7 | 0.018135 | Rutaceae | *Citrus* |
| 4.7 | 0.014662 | Araceae |  |
| 3.5 | 0.113591 | Rubiaceae |  |
| 3.5 | 0.105107 | Euphorbiaceae | *Alchornea* |
| 3.5 | 0.07984 | unknown |  |
| 3.5 | 0.059296 | Boraginaceae | *Cordia alba* |
| 3.5 | 0.045667 | unknown |  |
| 3.5 | 0.034797 | Solanaceae |  |
| 3.5 | 0.021468 | Apocynaceae |  |
| 3.5 | 0.010861 | unknown |  |
| 2.3 | 0.146756 | Melastomataceae |  |
| 2.3 | 0.03675 | unknown |  |
| 2.3 | 0.028447 | Lamiaceae |  |
| 2.3 | 0.018524 | unknown |  |
| 2.3 | 0.017442 | Balsaminaceae |  |
| 2.3 | 0.016594 | unknown |  |
| 2.3 | 0.015504 | Rosaceae |  |
| 2.3 | 0.01525 | Arecaceae |  |
| 2.3 | 0.01525 | Turneraceae |  |
| 2.3 | 9.56E-03 | unknown |  |
| 2.3 | 7.65E-03 | unknown |  |
| 2.3 | 7.53E-03 | Fabaceae | *Inga* |
| 2.3 | 7.48E-03 | Fabaceae | *Senna/Cassia* |
| 2.3 | 7.41E-03 | Acanthaceae | *Tricanthera gigantea* |
| 2.3 | 1.77E-03 | Acanthaceae | *Thunbergia* |
| 1.2 | 0.01458 | Sapindaceae |  |
| 1.2 | 0.013521 | unknown |  |
| 1.2 | 0.011805 | unknown |  |
| 1.2 | 9.92E-03 | Euphorbiaceae |  |
| 1.2 | 3.74E-03 | Asteraceae |  |
| 1.2 | 3.26E-03 | Boraginaceae | *Cordia alliodora* |
| 1.2 | 3.01E-03 | Boraginaceae | *Cordia spinescens* |
| 1.2 | 1.92E-03 | Asteraceae |  |
| 1.2 | 1.43E-03 | Solanaceae | *Cestrum* |
| 1.2 | 9.61E-04 | Sapindaceae |  |
| 1.2 | 8.34E-04 | unknown |  |


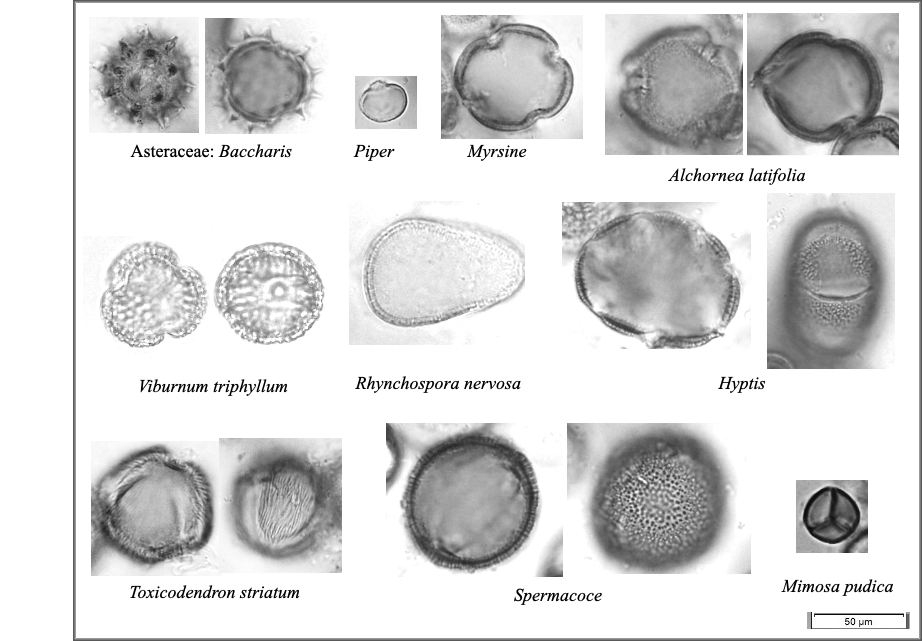


**Figure S2.** The ten most frequent pollen types found in *Tetragonisca angustula* beebread in Chameza, Casanare, CO.


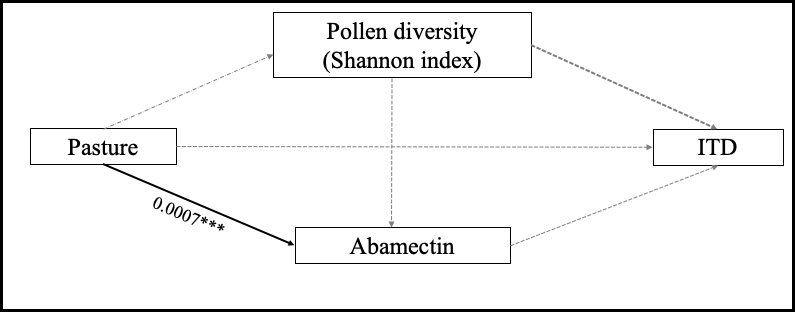


**Figure S3.** Path model of the relationships between the proportion of pasture in the landscape at 500m, Shannon diversity index, and the intertegular distance ITD (Fisher's C = 3.597 with P-value = 0.166 and on 2 degrees of freedom). Solid lines indicate significant effects, while dashed lines denote nonsignificant effects. Black lines are positive effects, while red lines are negative effects. The number along the arrows are the p-values and stars demark the significance level (*P < 0.05, **P < 0.01, ***P < 0.001).

**Table S4.** Acute toxicity values of Abamectin for *Tetragonisca angustula*

| Time (hours) | LC_50_ | C.I. 95% | X^2^ | D.F. |
| --- | --- | --- | --- | --- |
| 24 | 0.021 ug/ul | 0.017 - 0.027 | 87.047 | 25 |
| 48 | 0.019 ug/ul | 0.013 - 0.028 | 101.52 | 25 |

Mean lethal concentration (LC_50_), confidence interval 95% (C.I.), Chi-square (X^2^), Degree of freedom (D.F.).


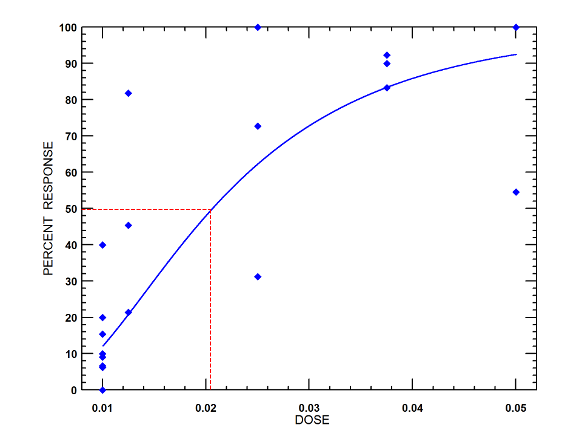


**Figure S5**. Abamectin LC50 probit calculation model for *Tetragonisca angustula*


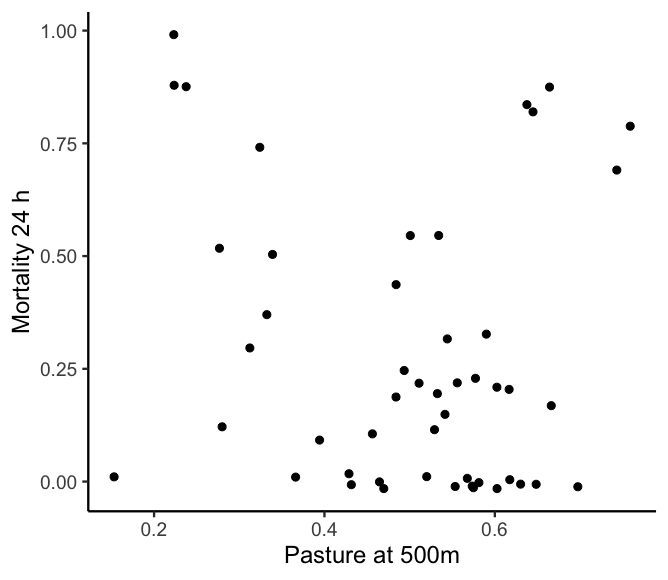


**Figure S6.** Mortality at 48 hours of *Tetragonisca angustula* worker bees fed with a solution of sugar-water containing a discriminatory concentration of abamectin based on the oral LC50 (0.021 ug/ul) in relation to the proportion of pasture area at 500m around the colonies.


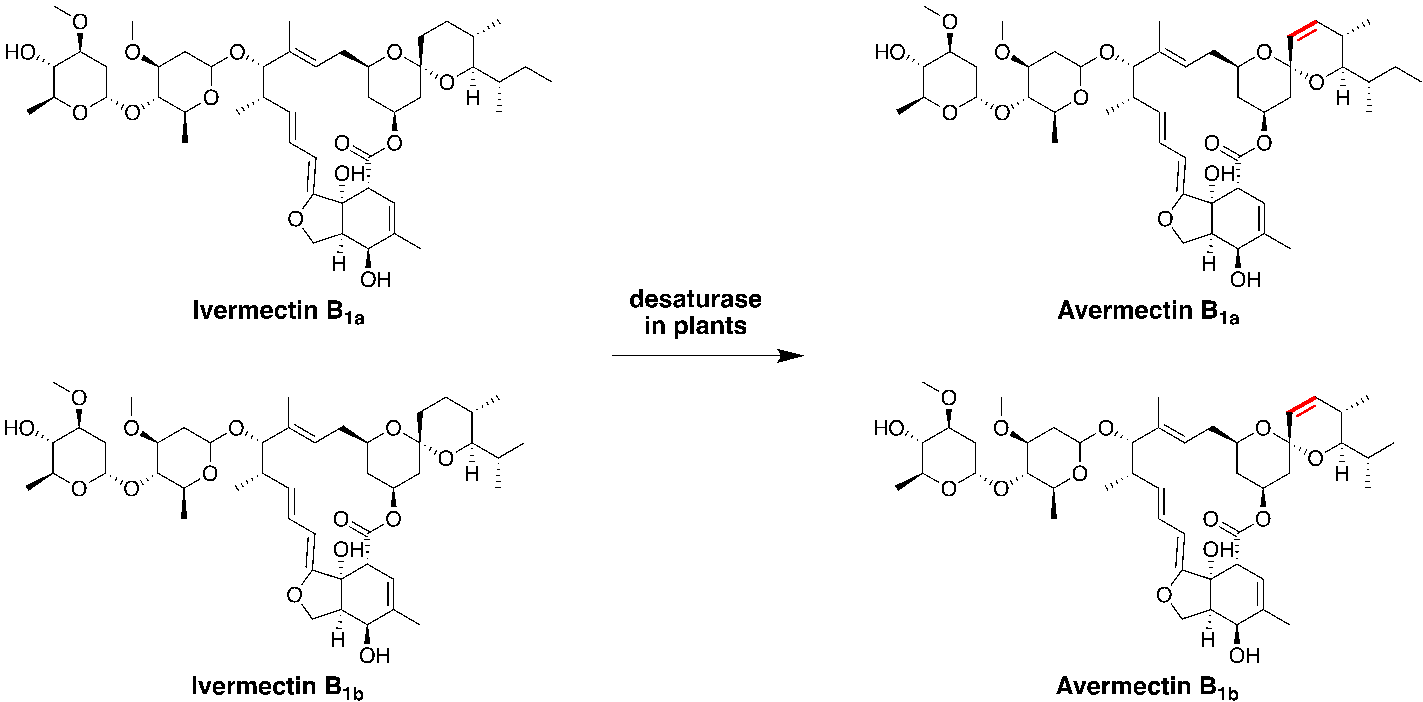


**Figure S7.** Proposed enzymatic transformation of ivermectin into abamectin in *Vernonanthura patens* highlighting in red the formation of the double bond*.*

**Table S8.** Abamectin LC50 bioassay:

To determine the abamectin oral LC50 for *Tetragonisca angustula,* we collected adult worker bees from 6 different hives, free from diseases and with a queen in good health, located in the stingless bee preserve AYNI in La Mesa, Cundinamarca, Colombia (4°41’39’’N, 74°25’48’’W). We selected a different study site from the landscape experiment to assure the bees came from a place with no pesticide exposure and >75% percent natural habitat in the landscape. Bees were randomly transferred in groups of 10 to 16 individuals to 150 ml deli cups and fed *ad libitum* with a 1.5 mL microcentrifuge tube feeder containing 500 uL of 50% sucrose with the respective abamectin treatment level. There were three replicates per treatment level for a total of 348 bees included in the bioassay.

We dissolved abamectin (Alfa Aesar 90.9% purity) in 100% dimethyl sulfoxide (DMSO), as suggested by the provider company and Poquet et al., 2014 (1), Guseman et al., 2016 (2) to reach an initial concentration of 1000ug/ml. This solution was diluted in 1:1 sucrose:water to a range of treatment levels and to obtain a final DMSO concentration < 5% (v/v). The treatment levels were selected using as a reference the oral LD50 for honey bees: 0.010 ug/bee and based on daily consumption of 2ul of solution per bee (3):

| Levels | ug/bee in 2ul of solution | Concentrations tested (ug/ul) |
| --- | --- | --- |
| 0x | 0 | 0 |
| -10x | 0.001 | 0.0005 |
| -7.5x | 0.001333 | 0.0007 |
| -5x | 0.002 | 0.001 |
| -2.5 X | 0.004 | 0.002 |
| 1X (LD50 for honey bees) | 0.01 | 0.005 |
| 2.5x | 0.025 | 0.0125 |
| 5x | 0.05 | 0.025 |
| 7.5x | 0.075 | 0.0375 |
| 10x | 0.1 | 0.05 |

Tested concentrations to calculate the oral abamectin LC50 in *Tetragonisca angustula* populations. The levels were selected based on the LD50 calculated for *Apis mellifera*.

Mortality was recorded as the proportion of individuals without movement after 24 and 48 hours. Probit analysis was used to estimate LC50 values, slopes, and 95% confidence intervals with the application POLO Probit (4). The goodness of fitness was tested with a Chi-square test.

**References of the supplementary information:**

1. Poquet, Y. *et al.* A pragmatic approach to assess the exposure of the honey bee (*Apis mellifera*) when subjected to pesticide spray. *PLoS One* **9**, (2014).
2. Guseman, A. J. *et al.* Multi-drug resistance transporters and a mechanism-based strategy for assessing risks of pesticide combinations to honey bees. *PLoS One* **11**, (2016).
3. del Sarto, M. C. L., Oliveira, E. E., Guedes, R. N. C. & Campos, L. A. O. Differential insecticide susceptibility of the Neotropical stingless bee *Melipona quadrifasciata* and the honey bee *Apis mellifera*. *Apidologie* (2014) doi:10.1007/s13592-014-0281-6.
4. Robertson, J., Russell, R. & Savin, N. POLO: Probit analysis. Preprint at (2014).
